# Variation in flower frost tolerance among seven apple cultivars and transcriptome response patterns in two contrastingly frost-tolerant selected cultivars

**DOI:** 10.1515/biol-2025-1107

**Published:** 2025-05-20

**Authors:** Je-Chang Lee, Mewuleddeg Zebro, Haet-Nim Jeong, Jae-Yun Heo

**Affiliations:** Department of Plant Science, Gangneung-Wonju National University, Juk-Heon Gil 7, Gangneung, 25457, Gangwon State, Korea; Horticulture Crops Research Unit, Gangwon State Agricultural Research and Extension Service, Chuncheon, 24203, Gangwon State, Korea

**Keywords:** climate change, differentially expressed genes, gene ontology, *Malus domestica*

## Abstract

This study evaluated frost tolerance in flowers of seven major apple cultivars grown in Korea to develop frost-resistant varieties for sustainable apple production under climate change. Flowers at full bloom were exposed to simulated frost conditions at −2°C, and frost damage was assessed using the total flower frost damage rate and King flower frost damage rate. Over 3 years, “Arisoo” consistently exhibited strong frost tolerance, whereas ‘Fuji’ was frost-sensitive. Transcriptomic analysis revealed significant differences in gene expression both within cultivars under different treatments and between cultivars under identical conditions. A higher number of differentially expressed genes were upregulated under frost stress in both cultivars, indicating key regulatory mechanisms involved in frost adaptation. Functional annotation and Kyoto Encyclopedia of Genes and Genome pathway analysis identified plant hormone signaling, mitogen-activated protein kinase signaling, and starch and sucrose metabolism can contribute to frost tolerance. Our findings offer critical insights into the genetic and molecular mechanisms of frost tolerance, contributing to the development of resilient apple varieties and sustainable production systems under climate change.

## 1 Introduction

Climate change has significantly increased the frequency and intensity of anomalous seasonal temperatures, disrupting the dormancy cycles of temperate fruit trees [1]. This disruption alters phenological development and exacerbates the susceptibility of reproductive tissues to spring frost damage [2]. In Korea, the progressive rise in mean temperatures has also led to an advancement in the apple flowering period [3], thereby exposing floral organs to unseasonal frost events. This heightened vulnerability has contributed to a nationwide increase in frost-induced damage to apple flowers. Since 2020, frost-related incidents have substantially reduced Korea’s average annual apple yield. In 2023, production declined sharply from 480,000 to 394,000 tons, representing a 30% decrease compared to the previous year, primarily due to frost damage during the critical bloom stage [4]. These significant yield losses have driven a sharp escalation in apple prices, underscoring the severe economic and agronomic ramifications of spring frost damage on the domestic apple industry.

In response to these challenges, extensive research has been conducted to develop frost mitigation strategies for apple orchards worldwide. Various protective measures have been explored, including sprinkler irrigation, where water freezes on the plant’s surface to form a protective layer of ice, and airflow disturbance technology, which enhances air circulation to prevent cold air stagnation [5,6]. While these methods have proven effective, their widespread adoption is limited by practical challenges, such as site-specific constraints and high installation and maintenance costs. Consequently, the implementation of these techniques in Korean apple orchards remains limited. Instead, most domestic apple growers rely on pre-bloom foliar sprays containing urea and magnesium to enhance frost resistance in apple flowers. However, this approach provides only partial and transient protection, underscoring the need for more effective and sustainable long-term solutions.

The development of frost-resistant apple cultivars represents a viable and sustainable strategy for mitigating frost damage in apple production. A previous study has categorized apple cultivars based on their susceptibility to frost events during flowering [7]. However, the predominant apple cultivars cultivated in Korea exhibit distinct genetic backgrounds and phenological characteristics compared to those analyzed in other regions. This variation underscores the necessity for region-specific evaluations of frost tolerance in apple flowers, as differences in genetic composition and local climatic conditions may influence cold stress responses.

Frost tolerance in apples is a complex polygenic trait governed by intricate interactions between genetic and environmental factors. Several genetic components associated with cold stress responses have been identified, with one of the most extensively studied regulatory pathways involving the Inducer of C-repeat binding factor (CBF) expression. This transcription factor modulates the expression of CBF genes, which, in turn, regulate cold-responsive (COR) genes, enhancing freezing tolerance at the molecular level [8]. Additionally, TIME FOR COFFEE has been implicated in protecting plants from freezing stress by promoting fatty acid unsaturation [9]. While these genetic pathways have been primarily characterized in apple leaves and woody tissues, their role in floral frost tolerance remains largely unexplored. This knowledge gap is particularly significant, as floral tissues exhibit distinct physiological and biochemical responses to cold stress compared to vegetative tissues. Given that floral survival directly influences fruit set and overall yield, elucidating the molecular mechanisms underlying flower frost tolerance is crucial for developing targeted strategies to mitigate frost-induced reproductive losses in apple production.

The present study aimed to assess the genetic variability in frost tolerance among apple flowers of cultivars widely grown in Korea. Cultivars exhibiting significant differences in frost resilience were subjected to transcriptome analysis to elucidate the key genetic and molecular mechanisms underlying frost tolerance. The findings of this research are essential foundational data for breeding programs focused on developing frost-resistant apple cultivars, specifically targeting flower frost tolerance. By integrating genetic insights with breeding strategies, this study offers a scientifically grounded and sustainable approach to mitigating the adverse effects of climate change on Korea’s apple industry. Ultimately, these efforts will contribute to securing the livelihoods of apple growers and ensuring the long-term stability of apple production in Korea.

## 2 Materials and methods

### 2.1 Sample collection and low-temperature treatment

This study was conducted from 2022 to 2024 at the Fruit Experiment Orchard of the Gangwon State Agricultural Research and Extension Services in Chuncheon, Gangwon State, Korea. The experiment targeted four domestically developed apple cultivars (“Hongro,” “Honggeum,” “Arisoo,” and “Picnic”), which are predominantly cultivated in the Gangwon state, along with three major introduced cultivars (‘Fuji,’ “Tsugaru,” and “Sinano Gold”). All cultivars were grafted onto M.9 apple rootstocks and were 5 years old as of 2022. To ensure consistency in experimental conditions, the fruit load was standardized to three fruits per trunk cross-sectional area for each tree. Furthermore, uniform fertilization, pest management, and other cultivation practices were applied to minimize external variability and ensure reliable results. For each cultivar, flower samples were collected from clusters of five flowers (one flower cluster) that had developed from terminal buds located at the upper part of fruiting spurs. The spurs were approximately 3.5 mm in diameter and 20 cm in length. Sampling was carried out during the full bloom stage, defined as the point when all flowers in the cluster were fully open. To guarantee sufficient representation, 12 fruiting spurs were sampled for each cultivar.

Low-temperature treatments were carefully conducted using a high-precision refrigerated thermo-hygrostat (Jeio-Tech, Daejeon, Korea) in the postharvest management building of the Gangwon State Agricultural Research and Extension Services. To replicate realistic frost conditions, the minimum treatment temperature was set at −2°C, as this is the critical threshold for frost damage in blooming apple flowers. The treatment process began with the thermo-hygrostat set at 10°C. The temperature was then gradually lowered at a controlled rate of 2°C per hour until it reached −2°C. The samples were maintained at −2°C for 4 h before the temperature was gradually increased at the same rate back to 10°C. After the temperature adjustment, the samples were left at room temperature for 12 h to allow for consistent recovery. Following this recovery period, the frost damage assessment was conducted.

### 2.2 Evaluation of flower frost tolerance among apple cultivars

To evaluate the extent of frost damage, flowers were carefully dissected, and the cross-sections were examined. Frost damage was determined by the browning of the ovary or pistil, which was regarded as a clear indication of cell death. Recognizing the unique corymb flowering structure of apples, frost damage was quantified using two distinct metrics: the total flower frost damage rate (TFFD rate), representing the frost damage across all flowers in the cluster, and the king flower frost damage rate (KFFD rate), focusing on the commercially significant central (king) flower. The TFFD rate was calculated as the percentage of frost-damaged flowers out of the total number of flowers in the cluster, while the KFFD rate was calculated as the percentage of frost-damaged king flowers out of the total number of king flowers in all clusters. These metrics provide a comprehensive understanding of the extent of frost damage and its potential impact on apple production.

The calculations are as follows:

\[\text{Total flower frost damage rate}\hspace{.5em}( \% )=(\text{Number of frost}-\text{damaged flowers}/\text{Total number of flowers})\times 100,]\]

\[\text{King flower frost damage rate}\hspace{.5em}( \% )=(\text{Number of frost}-\text{damaged king flowers}/\text{Total number of king flowers})\times 100.]\]

### 2.3 RNA extraction, sequencing, and functional annotation

In this study, we selected two apple cultivars with differing frost tolerances in flowers. To analyze their response to frost stress, samples were collected from plants exposed to −2°C for 4 h during the temperature treatment process used for phenotypic evaluation. Untreated control samples were maintained at room temperature without frost exposure. All samples designated for transcriptome analysis were immediately stored at −80°C to preserve RNA integrity. RNA was extracted from 100 mg of each frozen sample using the RNeasy Plant Mini Kit (QIAGEN, CA, USA), following the manufacturer’s protocol. Genomic DNA contamination was eliminated through DNaseI treatment (QIAGEN, CA, USA). The concentration of total RNA was quantified using the Quant-IT RiboGreen assay (Invitrogen, MA, USA), and quality control (QC) was performed using the RNA ScreenTape assay on the TapeStation system (Agilent Technologies, CA, USA). Only RNA samples with a RIN (RNA integrity number) of 6.0 or higher were used for library preparation. For RNA library construction, 1 µg of total RNA per sample was utilized. Libraries were prepared using the NEXTFLEX^®^ Rapid Directional RNA-Seq Kit 2.0 (Revvity Health Sciences, MA, USA), which included RNA fragmentation followed by reverse transcription to synthesize the first-strand complementary DNA (cDNA). Subsequent second-strand synthesis was performed to complete the cDNA. Single “A” base addition and adapter ligation were then carried out to prepare the final cDNA libraries. Library QC was assessed using the TapeStation D1000 ScreenTape assay (Agilent Technologies, CA, USA). Libraries that met QC criteria were sequenced using paired-end reads (2 × 151 bp) on the Illumina NovaSeq platform (Illumina, Inc., CA, USA). Adaptor sequences were trimmed from the raw reads using Trimmomatic (v0.39). The clean reads were aligned to the *Malus* × *domestica* HFTH1 Whole Genome v1.0 using HISAT2. Gene expression levels were quantified by calculating the total number of reads mapped to each gene, utilizing HTSeq (v0.11.0). Differentially expressed genes (DEGs) were identified using DESeq2 with thresholds of a fold change ≥2 and a divergence probability of 0.01 or less. Functional annotation and pathway analysis of DEGs were performed using Gene Ontology (GO) and Kyoto Encyclopedia of Genes and Genomes (KEGG) tools.

### 2.4 Quantitative real-time PCR (qRT-PCR) analysis for confirmation

To confirm the transcriptomic data, total RNA was extracted using the Takara MiniBEST Plant RNA Extraction Kit (Takara Bio Inc., Shiga, Japan), and cDNA was synthesized using Primer Script™ RT Reagent Kit, which includes a gDNA removal step for eliminating genomic DNA contamination (Takara Bio Inc., Shiga, Japan). qRT-PCR was performed using the TB Green^®^ Premix Ex Taq™ II (Tli RNaseH Plus). The reaction mixture was prepared to a total volume of 25 μL, which included 3 μL of cDNA as the template, 5 μL of a primer mix (2.5 μL each of forward and reverse primers), 4.5 μL of nuclease-free water, and 12.5 μL of TB Green PCR Master Mix. The qPCR cycling protocol began with an initial denaturation at 95°C for 5 min, followed by 35 amplification cycles at 95°C for 30 s, 55°C for 40 s, and 72°C for 1 min. To assess the specificity of amplification and detect potential nonspecific products, a melting curve analysis was performed at the end of the run, with temperatures incrementally increased from 65 to 95°C. Primers were designed using the Primer3Plus software tool (https://www.primer3plus.com/index.html). The *Mdactin* gene was utilized as the internal control for normalization. Gene expression levels were quantified using the 2^−ΔΔ*C*^_T_ method, and each sample was analyzed with three biological replicates.

### 2.5 Statistical analysis

Statistical analysis was performed using R software version 4.4.1 (R Foundation for Statistical Computing, Vienna, Austria). Data were collected in triplicates, and the results were expressed as mean values with standard deviations. A two-way analysis of variance (two-way ANOVA) was conducted to evaluate the effects of cultivar types and experimental years on the frost damage rate of apple flowers under low-temperature treatment. Post-hoc analysis was carried out using Duncan’s multiple range test, and statistical significance was considered at a *p*-value ≤0.05. Additionally, Tukey’s honest significant difference (HSD) test was performed to further analyze differences in frost tolerance among specific cultivars.

## 3 Results

### 3.1 Evaluation of flower frost tolerance among apple cultivars

**Figure 1:**
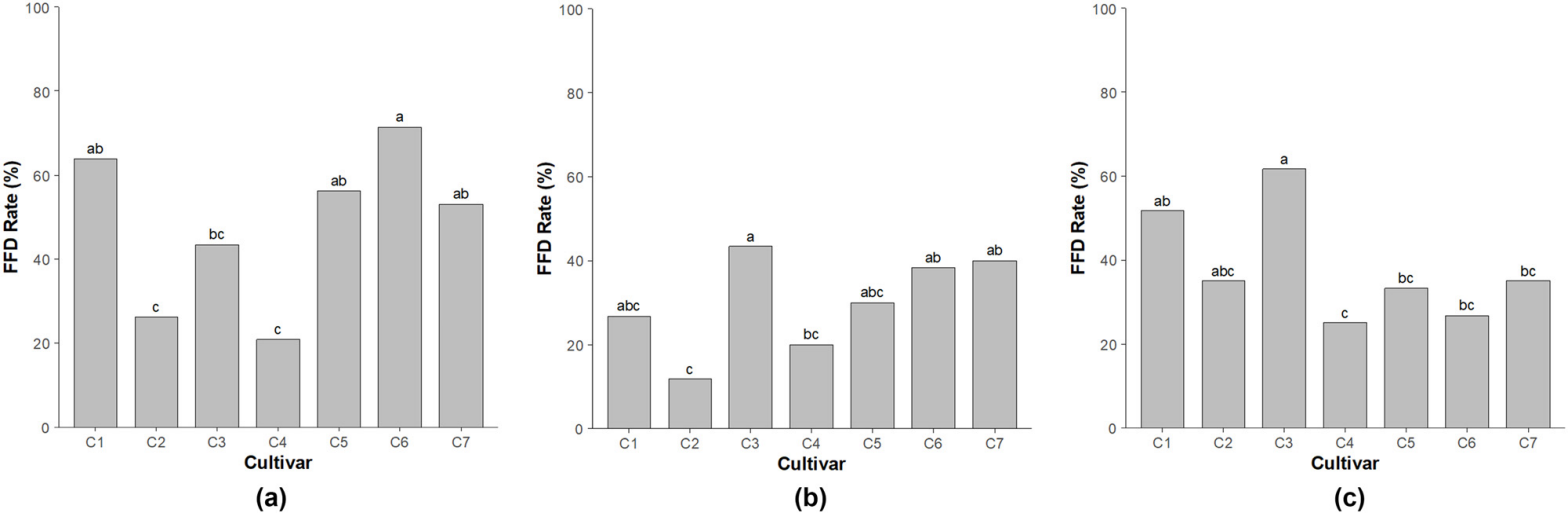
TFFD rates following artificial low-temperature treatment for major apple cultivars grown in the Gangwon region of Korea: (a) 2022, (b) 2023, and (c) 2024. ^y^Different letters indicate the significant differences among the mean values at *p* < 0.05, ^z^C1: ‘Fuji’; C2: “Hongro”; C3: “Honggeum”; C4: “Arisoo”; C5: “SinanoGold”; C6: “Tsugaru”; C7: “Picnic.”

To evaluate frost tolerance in apple flowers during spring, year-by-year variations in frost damage were assessed using artificial low-temperature treatments. Statistical analysis through ANOVA revealed significant differences in frost tolerance among seven apple cultivars across 3 years of observations and in the interactions between these factors (Table 1). The TFFD rate demonstrated clear and distinct trends across cultivars and years (Figure 1). In 2022, TFFD rates ranged from 20.9 to 71.4%, with “Hongro” and “Arisoo” exhibiting the lowest damage rates at 26.2 and 20.9%, respectively, while “Tsugaru” showed the highest damage rate at 71.4%. Other cultivars, including ‘Fuji,’ “Sinano Gold,” and “Picnic,” also exhibited relatively high frost damage rates. In 2023, TFFD rates ranged from 11.7 to 43.3%, with “Hongro” showing the lowest damage rate (11.7%) and “Honggeum” the highest (43.3%). By 2024, TFFD rates ranged from 25.0 to 61.7%, with “Arisoo” once again demonstrating the lowest damage rate (25.0%), while ‘Fuji’ and “Honggeum” recorded higher rates of 51.7 and 61.7%, respectively. Statistically significant differences in TFFD rates were observed between 2022 and 2023 at a 95% confidence level; however, no significant differences were observed between 2022–2024 and 2023–2024 (data not shown).

**Table 1:** ANOVA results for flower frost damage rates by cultivars and years

Variable	Cultivar (*C*)	Year (*Y*)	*C* × *Y*
**Source of variation**			
TFFD rate	6.963***	11.144***	3.153**
KFFD rate	4.561**	6.705**	2.454*

***, **, *: significance levels at *p* < 0.001, *p* < 0.01, and *p* < 0.05, respectively. The values for the TFFD rate and KFFD rate are expressed as percentages (%). TFFD rate: total flower frost damage rate; KFFD rate: king flower frost damage rate.

**Figure 2:**
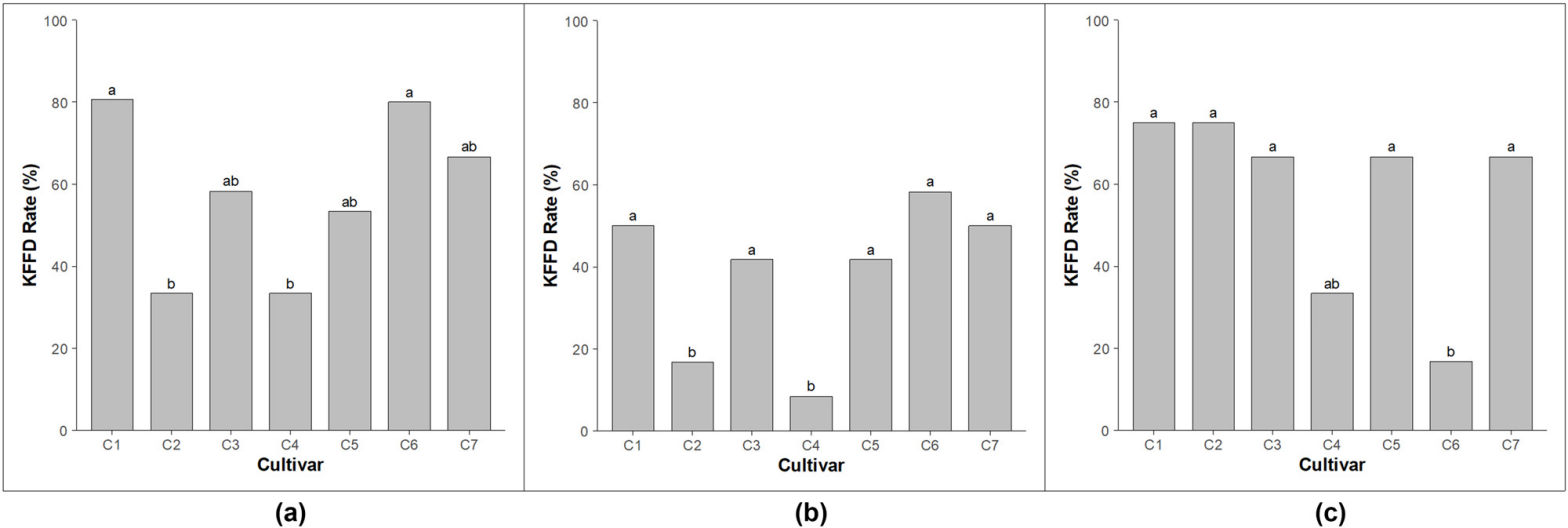
Annual KFFD following artificial low-temperature treatment for major apple cultivars grown in the Gangwon region of Korea: (a) 2022, (b) 2023, and (c) 2024. ^y^Different letters indicate the significant differences among the mean values at *p* < 0.05, ^z^C1: ‘Fuji’; C2: “Hongro”; C3: “Honggeum”; C4: “Arisoo”; C5: “SinanoGold”; C6: “Tsugaru”; C7: “Picnic.”

The KFFD rate was consistently higher than the TFFD rate, likely because king flowers, which develop earlier than other flowers in the cluster, are more susceptible to frost damage under identical low-temperature conditions. In 2022, KFFD rates ranged from 33.3 to 80.6%, with “Hongro” and “Arisoo” showing the lowest rates (33.3%), while ‘Fuji’ and “Tsugaru” recorded the highest rates at 80.6 and 80.0%, respectively, mirroring trends observed in the TFFD rates (Figure 2). In 2023, KFFD rates ranged from 8.3 to 58.3%, with damage rates exceeding 41.7% in all cultivars except “Hongro” and “Arisoo.” By 2024, “Tsugaru” exhibited the lowest KFFD rate (16.7%), while all other cultivars except “Arisoo” exhibited rates exceeding 66.7%. Statistically significant differences were identified between 2022 and 2023, as well as between 2023 and 2024, at a 95% confidence level; however, no significant differences were observed between 2022 and 2024 (data not shown).

The potential application of these cultivars as breeding materials for developing frost-resistant apple varieties was also evaluated. Pairwise differences among cultivars were analyzed using Tukey’s HSD test. Based on TFFD rates across 3 years, significant differences were identified in 9 of the 21 pairwise comparisons (Table 2). Among these, the combination of “Honggeum” and “Arisoo” exhibited the most significant difference (*P* < 0.001), while combinations such as ‘Fuji’–“Arisoo” and ‘Fuji’–“Hongro” also showed significant differences (*P* < 0.01). These results were consistent with the trends observed in Figure 1. Similarly, based on KFFD rates over 3 years, significant differences were observed in 3 of the 21 pairwise comparisons (Table 3). The combination of ‘Fuji’ and “Arisoo” demonstrated the most significant difference (*P* < 0.001), while the “Tsugaru”–“Arisoo” combination also showed significant differences (*P* < 0.01).

**Table 2:** Comparison of TFFD rates among major apple cultivars grown in Gangwon State, Korea

Comparison	Mean Diff.	Lower CI	Upper CI	*P*-value	Significance
‘Fuji’–“Sinano Gold”	7.6000	−10.2955	25.4955	0.8413	NS
‘Fuji’–“Tsugaru”	1.9889	−15.9066	19.8844	0.9999	NS
‘Fuji’–“Arisoo”	25.5000	7.6045	43.3955	0.0013	**
‘Fuji’–“Picnic”	4.7667	−13.1288	22.6622	0.9810	NS
‘Fuji’–“Hongro”	21.5111	3.6156	39.4066	0.0097	**
‘Fuji’–“Honggeum”	−2.0111	−19.9066	15.8844	0.9998	NS
“Hongro”–“Sinano Gold”	−13.9111	−31.8066	3.9844	0.2215	NS
“Hongro”–“Tsugaru”	−19.5222	−37.4177	−1.6267	0.0246	*
“Hongro”–“Arisoo”	3.9889	−13.9066	21.8844	0.9925	NS
“Hongro”–“Picnic”	−16.7444	−34.6399	1.1510	0.0800	NS
“Hongro”–“Honggeum”	−23.5222	−41.4177	−5.6267	0.0036	**
“Honggeum”–“Sinano Gold”	9.6111	−8.2844	27.5066	0.6437	NS
“Honggeum”–“Tsugaru”	4.0000	−13.8955	21.8955	0.9924	NS
“Honggeum”–“Arisoo”	27.5111	9.6156	45.4066	0.0004	***
“Honggeum”–“Picnic”	6.7778	−11.1177	24.6733	0.9005	NS
“Picnic”–“Arisoo”	20.7333	2.8378	38.6288	0.0140	*
“Picnic”–“Sinano Gold”	2.8333	−15.0622	20.7288	0.9989	NS
“Picnic”–“Tsugaru”	−2.7778	−20.6733	15.1177	0.9990	NS
“Arisoo”–“Sinano Gold”	−17.9000	−35.7955	−0.0045	0.0499	*
“Arisoo”–“Tsugaru”	−23.5111	−41.4066	−5.6156	0.0036	**
“Tsugaru”–“Sinano Gold”	5.6111	−12.2844	23.0566	0.9576	NS

***, **, *: significance levels at *p* < 0.001, *p* < 0.01, and *p* < 0.05, respectively. NS: non-significant; Mean Diff.: mean difference; Lower CI: lower confidence interval; Upper CI: upper confidence interval.

**Table 3:** Comparison of KFFD rates among major apple cultivars grown in Gangwon State, Korea

Comparison	Mean Diff.	Lower CI	Upper CI	*P*-value	Significance
‘Fuji’–“Sinano Gold”	14.6333	−10.2955	43.6450	0.7067	NS
‘Fuji’–“Tsugaru”	16.8556	−12.1561	45.8672	0.5564	NS
‘Fuji’–“Arisoo”	43.5222	14.5106	72.5339	0.0006	***
‘Fuji’–“Picnic”	7.4111	−21.6005	36.4228	0.9846	NS
‘Fuji’–“Hongro”	12.9667	−16.0450	41.9783	0.8073	NS
‘Fuji’–“Honggeum”	26.8556	−2.1561	55.8672	0.0858	NS
“Hongro”–“Sinano Gold”	−12.2222	−31.8066	3.9844	0.8462	NS
“Hongro”–“Tsugaru”	−10.0000	−39.0117	19.0117	0.9342	NS
“Hongro”–“Arisoo”	16.6667	−12.3450	45.6783	0.5694	NS
“Hongro”–“Picnic”	−19.4444	−48.4561	9.5672	0.3858	NS
“Hongro”–“Honggeum”	−13.8889	−42.9005	15.1228	0.7536	NS
“Honggeum”–“Sinano Gold”	1.6667	−27.3450	30.6783	1.0000	NS
“Honggeum”–“Tsugaru”	3.8889	−25.1228	32.9005	0.9996	NS
“Honggeum”–“Arisoo”	30.5556	1.5439	59.5672	0.0332	*
“Honggeum”–“Picnic”	−5.5556	−34.5672	23.4561	0.9967	NS
“Picnic”–“Arisoo”	−28.8889	−57.9005	0.1228	0.0516	NS
“Picnic”–“Sinano Gold”	−26.6667	−55.6783	2.3450	0.0898	NS
“Picnic”–“Tsugaru”	7.2222	−21.7894	36.2339	0.9866	NS
“Arisoo”–“Sinano Gold”	9.4444	−19.5672	38.4561	0.9494	NS
“Arisoo”–“Tsugaru”	36.1111	7.0995	65.1228	0.0067	**
“Tsugaru”–“Sinano Gold”	−2.2222	−31.2339	26.7894	1.0000	NS

***, **, *: significance levels at *p* < 0.001, *p* < 0.01, and *p* < 0.05, respectively. NS: non-significant; Mean Diff.: mean difference; Lower CI: lower confidence interval; Upper CI: upper confidence interval.

“Arisoo” was selected for further experiments due to its exceptional fruit quality, stable coloration under high-temperature conditions, and expanding cultivation area in Korea. As a purebred cultivar, it is harvested during the Chuseok holiday season, a period of peak market value for apples, making it a commercially significant variety. Conversely, ‘Fuji’ represents over 60% of Korea’s total apple production and is widely preferred by consumers due to its outstanding storability and favorable fruit attributes, enabling prolonged market availability. The selection of these two cultivars is strategically important, as their distinct genetic and agronomic traits provide valuable insights for breeding programs and fundamental research aimed at improving apple resilience and quality in response to climate change.

### 3.2 RNA-seq data sequence statistics and function annotation for unigenes

To investigate the gene expression profiles of the apple cultivars “Arisoo” and ‘Fuji’ under both non-stress (control) and frost stress conditions, transcriptome sequencing was performed using the Illumina NovaSeq platform (Illumina, Inc., CA, USA). After removing contaminants and adaptors, the sequencing generated 154.08 and 150.84 Mb of raw data for “Arisoo” and ‘Fuji,’ respectively. Following filtering, each sample retained approximately 25.41 Mb of clean data, with a Q30 base percentage of 93.63% and a GC content of 46.00%. The mapping rate ranged from 95.34 to 96.42% (Table 4). A total of 44,677 unigenes were identified, with an average length of 1,180 bp, a GC content of 45.92%, and an N50 value of 1,719 bp. The majority of unigenes (28.66%) ranged from 200 to 500 bp in length. The longest unigene measured 17,340 bp, while the shortest was 21 bp. The assembled unigenes were annotated using multiple public databases, including NR, Swiss-Prot, TrEMBL, KEGG, GO, and TAIR10. The annotation rates were 88.08% for NR, 67.65% for Swiss-Prot, 86.49% for TrEMBL, 15.63% for KEGG, 53.19% for GO, and 77.60% for TAIR10 (Table 5).

**Table 4:** Sequence statistics of apple transcriptome

Sample name	Total raw reads	Total clean data	Clean bases (bp)	Total mapped	Mapping rate (%)	Expressed gene	Clean reads Q20 (%)	Clean reads Q30 (%)	GC%
AR-FS	24,935,041	24,104,924	7,108,814,153	23,075,777	95.73	21,453	98.13	93.70	46.04
AR-CK	25,759,176	25,060,810	7,390,609,525	24,163,310	96.42	21,412	98.13	93.69	45.96
FJ-FS	24,450,590	23,781,468	7,001,900,237	23,010,482	95.76	21,386	98.08	93.58	45.68
FJ-CK	25,830,255	24,971,026	7,317,097,513	23,807,767	95.34	21,581	98.07	93.56	46.33

**Table 5:** Unigenes annotated database and unigene length distribution

Database	Annotated number	Annotated ratio (%)
GO	23,765	53.19%
KEGG	6,986	15.63%
NR	39,353	88.08
TAIR10	34,671	77.60
SwissProt	30,223	67.65
TrEMBL	38,639	86.49

Unigenes	Number	Percentage
200–500 bp length	12,805	28.66
500–1,000 bp length	11,464	25.66
1,000–2,000 bp length	12,488	27.95
>2,000 bp length	7,052	15.78
Total	44,677	100.00
Minimum length (bp)	21	—
Mean length (bp)	1,180	—
Maximum length (bp)	17,340	—
N50	1,719	—
N90	873	—
GC (%)	—	45.92

### 3.3 DEG analysis

**Figure 3:**
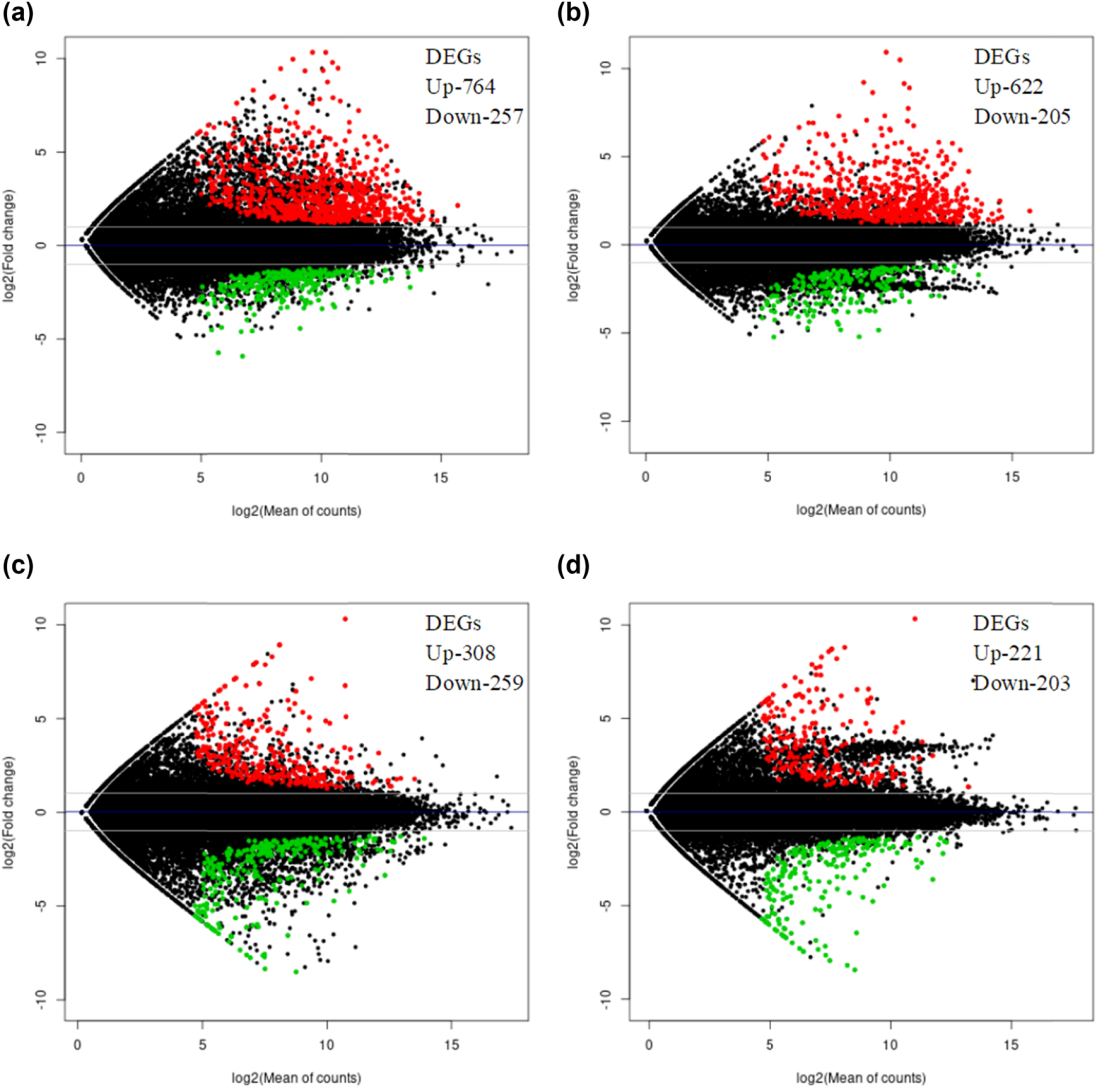
The MA plot illustrating the DEGs of the four comparison groups of two apple cultivars under cold stress conditions (a: AR-FS vs AR-CK, b: FJ-FS vs FJ-CK, c: FJ-FS vs AR-FS, and d: FJ-CK vs AR-CK). AR-FS: “Arisoo” frost stress, AR-CK: “Arisoo” control, FJ-FS: ‘Fuji’ frost stress and FJ-CK: ‘Fuji’ control, red and green colors indicate the up- and downregulated DEGs, respectively.

**Figure 4:**
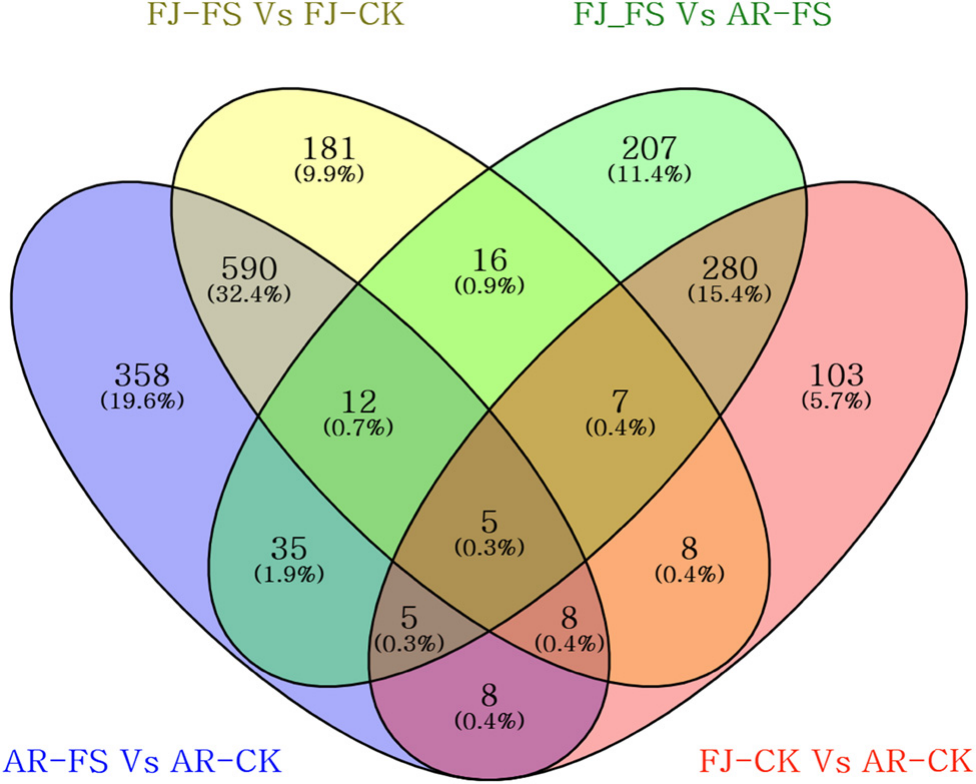
Venn diagrams illustrating the unique DEGs for the four comparison groups. AR-FS: “Arisoo” frost stress, AR-CK: “Arisoo” control, FJ-FS: ‘Fuji’ frost stress, and FJ-CK: ‘Fuji’ control, red and green colors indicate the up- and downregulated DEGs, respectively.

In this study, we quantitatively analyzed gene expression levels to identify DEGs between two apple cultivars: “Arisoo” (AR) and ‘Fuji’ (FJ). We compared the same cultivar under different conditions (control, CK, and frost stress, FS), as well as different cultivars under the same conditions. This resulted in four comparison groups: AR-FS vs AR-CK, FJ-FS vs FJ-CK, FJ-FS vs AR-FS, and FJ-CK vs AR-CK. Our analysis revealed a significant number of DEGs in each comparison group. In the AR-FS vs AR-CK group, there were 1,021 DEGs, with 764 upregulated and 257 downregulated (Figure 3a). In the FJ-FS vs FJ-CK group, there were 828 DEGs, with 622 upregulated and 205 downregulated (Figure 3b). When comparing different cultivars under the same conditions (FJ-FS vs AR-FS group), there were 567 DEGs, with 308 upregulated and 259 downregulated (Figure 3c). Similarly, in the FJ-CK vs AR-CK group, there were 424 DEGs, with 221 upregulated and 203 downregulated (Figure 3d). In addition, unique DEGs were identified across the four comparison groups. Figure 4 presents a Venn diagram illustrating these unique DEGs, with a total of 1,823. These DEGs were divided into 15 distinct groups. Specifically, 19.6, 11.4, 9.9, and 5.7% of the DEGs were unique to the comparisons AR-FS vs AR-CK, FJ-FS vs FJ-CK, FJ-FS vs AR-FS, and FJ-CK vs AR-CK, respectively. Furthermore, five DEGs (HF40776-RA, HF32305-RA, HF05616-RA, HF36100-RA, and HF32319-RA) were found to be commonly expressed across all groups. These findings indicate significant variations in gene expression both within cultivars under different treatments and between cultivars under the same conditions. Furthermore, there is a higher number of upregulated DEGs observed in the frost stress conditions for both cultivars.

### 3.4 GO enrichment analysis

**Figure 5:**
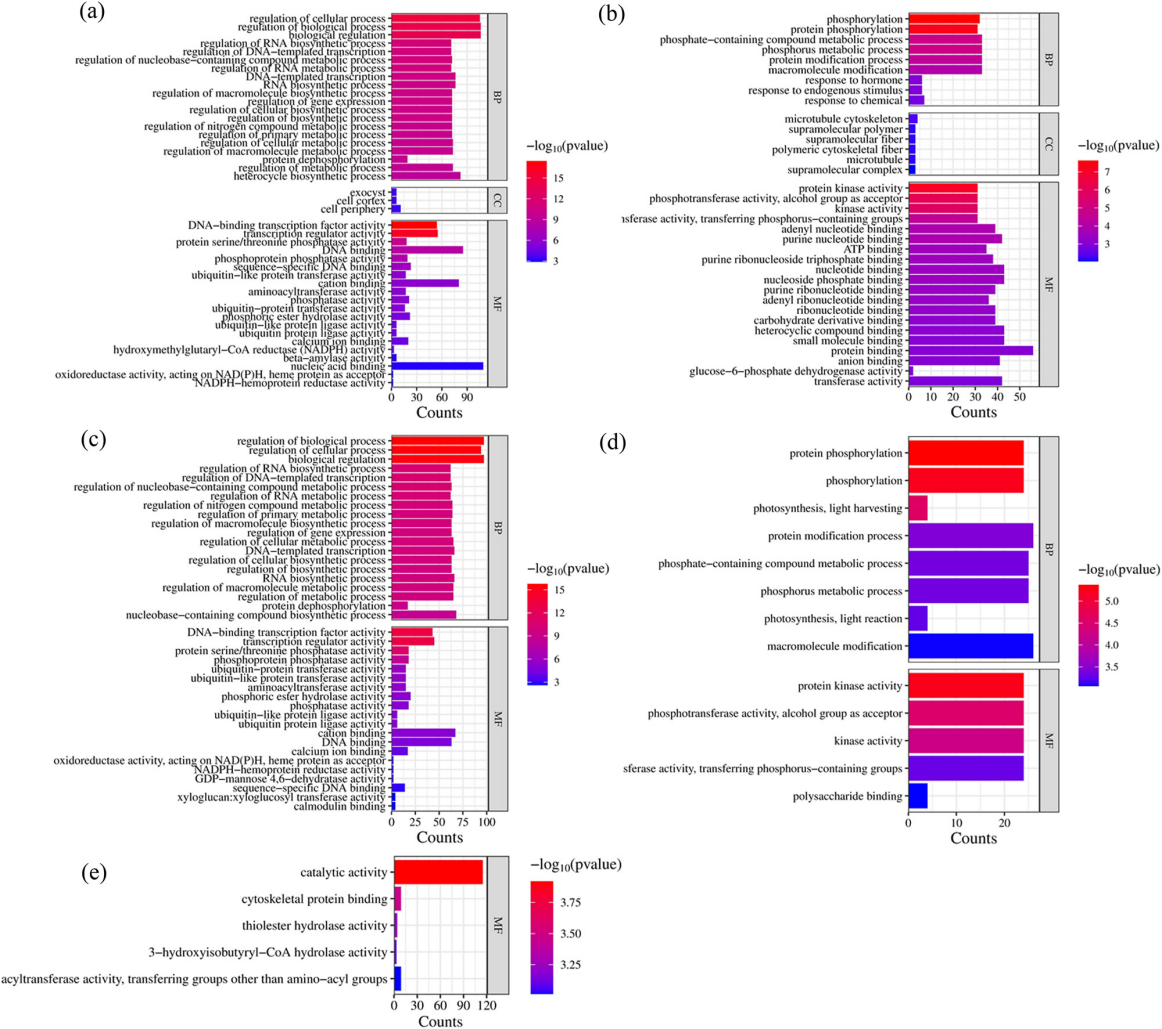
The top 20 significantly enriched GO terms of two apple cultivars in response to cold temperatures. (a) and (b) Up- and downregulated DEGs profile of AR-FS vs AR-CK, (c) and (d) up- and downregulated DEGs profile between FJ-FS vs FJ-CK, and (e) upregulated DEGs at FJ-FS vs AR-FS. AR-FS: “Arisoo” frost stress, AR-CK: “Arisoo” control, FJ-FS: ‘Fuji’ frost stress, and FJ-CK: ‘Fuji’ control, red and green colors indicate the up- and downregulated DEGs, respectively.

The GO classification and enrichment analysis of DEGs in the apple cultivars Arisoo and Fuji under frost stress reveal distinct adaptive patterns. The top 20 significantly enriched GO classifications are presented in Figure 5. In the AR-FS vs AR-CK comparison group, upregulated transcripts are associated with various biological processes, molecular functions, and cellular components. Notable biological processes include the regulation of biological and cellular processes as well as biological regulation (Figure 5a). Molecular functions include nucleic acid binding, cation binding, DNA binding, transcription regulator activity, and DNA binding transcription factor activity (Figure 5a). Cellular components include the cell periphery, exocyst, and cell cortex (Figure 5a). The most prominent categories among downregulated AR-FS vs AR-CK transcripts are macromolecule modification, protein modification process, phosphorus metabolic process, and phosphorylation, indicating a reduction in specific signaling pathways and protein modification mechanisms to conserve energy during frost stress (Figure 5b). In the FJ-FS vs FJ-CK comparison group, upregulated transcripts primarily relate to the regulation of biological and cellular processes, indicating a dynamic response involving various regulatory pathways (Figure 5c). Key molecular functions include cation binding, DNA binding, transcription regulator activity, and DNA binding transcription factor activity, suggesting significant changes in gene expression to manage frost stress effects (Figure 5c). Downregulated transcripts are mainly associated with protein modification processes and macromolecule modification, showing a shift in cellular metabolic activities to adapt to the frost stress condition (Figure 5d). When comparing both cultivars under frost stress (FJ-FS vs AR-FS), the significant GO category detected was molecular function, with upregulated transcripts mainly representing catalytic activity (Figure 5e). This suggests that the primary differences in response to frost stress between the two cultivars involve the activation of enzymes and catalytic proteins, potentially playing a crucial role in frost tolerance through accelerated biosynthesis of protective compounds and activation of stress-mitigating metabolic pathways. No significantly enriched GO terms were found for downregulated transcripts in this comparison. Under control conditions (FJ-CK vs AR-CK), molecular function was also the significant GO category detected, and catalytic activity was the most represented among upregulated transcripts. This suggests inherent differences in metabolic activities between the two cultivars under normal conditions, which may be linked to their frost tolerance capabilities. Once again, no significantly enriched GO terms were found for downregulated transcripts, highlighting that functional differences under control conditions are primarily characterized by upregulated catalytic activities.

### 3.5 KEGG pathway analysis

**Figure 6:**
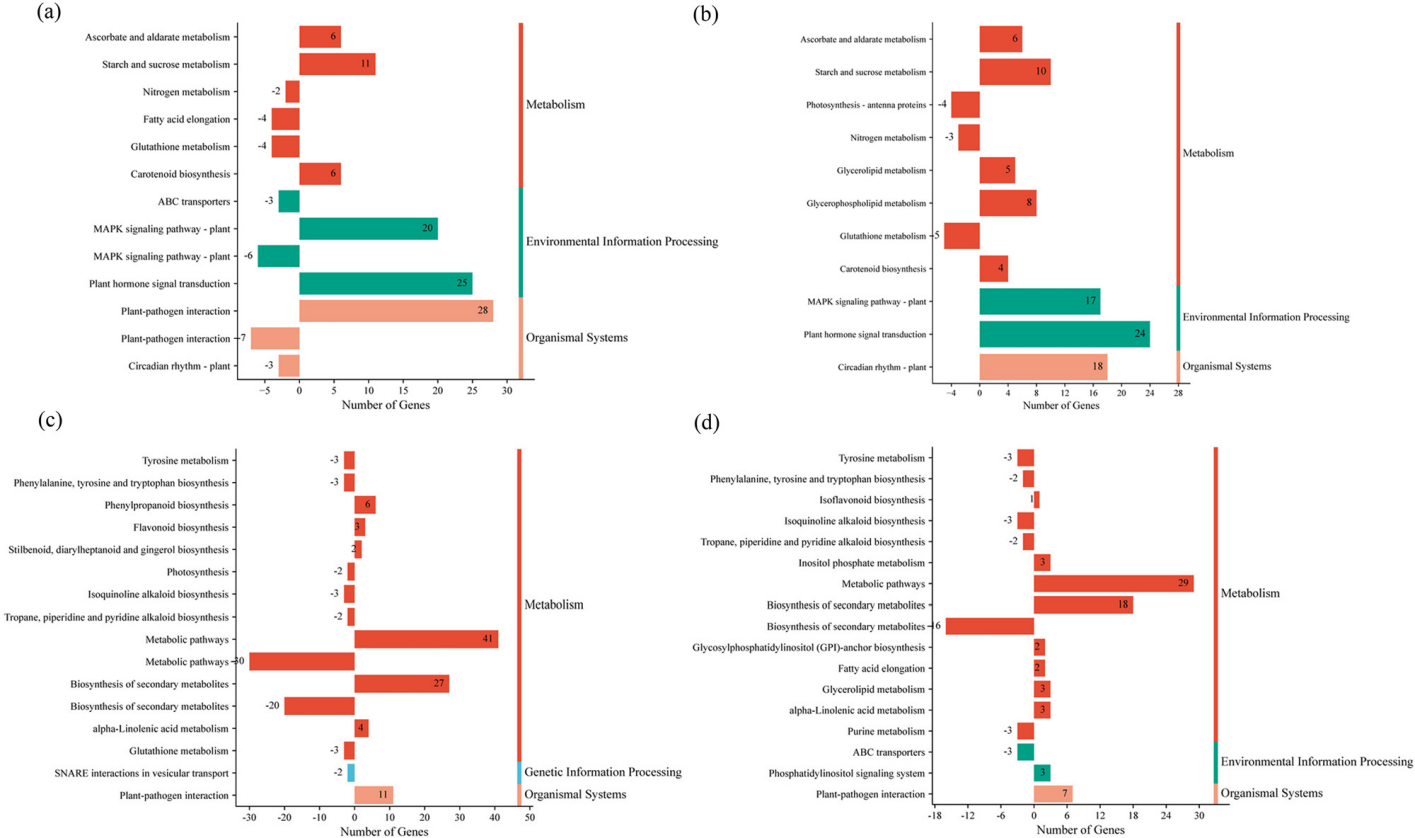
Bar graphs illustrating the KEGG pathway that is significantly enriched under cold stress conditions in the Arisoo and Fuji apple cultivars. (a)–(d) Downregulated and upregulated pathways in the AR-FS vs AR-CK, FJ-FS vs FJ-CK, FJ-FS vs AR-FS, and FJ-CK vs AR-CK comparison groups, respectively. The negative sign on the *X*-axis indicates the number of downregulated DEGs. AR-FS: “Arisoo” frost stress, AR-CK: “Arisoo” control, FJ-FS: ‘Fuji’ frost stress, and FJ-CK: ‘Fuji’ control, red and green colors indicate the up- and downregulated DEGs, respectively.

A pathway-based analysis using the KEGG pathway database was conducted to further explore the DEGs involved in various metabolic pathways. The KEGG pathway analysis identified several significantly enriched pathways across four comparison groups (Figure 6). In the metabolism category, commonly upregulated pathways in both the AR-FS vs AR-CK and FJ-FS vs FJ-CK comparisons included ascorbate and aldarate metabolism, starch and sucrose metabolism, and carotenoid biosynthesis (Figure 6a and b). Conversely, nitrogen metabolism and glutathione metabolism pathways were consistently downregulated in these groups (Figure 6a and b). Additionally, in the AR-FS vs AR-CK comparison, the fatty acid elongation pathway was downregulated (Figure 3a), while in the FJ-FS vs FJ-CK comparison, glycerolipid metabolism, and glycerophospholipid metabolism pathways were upregulated, and the photosynthesis–antenna protein pathway was downregulated (Figure 6b). In the environmental information processing category, the plant hormone signal transduction and mitogen-activated protein kinase (MAPK) signaling pathways were commonly enriched and upregulated in both the AR-FS vs AR-CK and FJ-FS vs FJ-CK comparisons (Figure 6a and b). Notably, a few DEGs related to the MAPK signaling pathway and ABC transporters were downregulated in the AR-FS vs AR-CK comparison (Figure 6a). Under the organismal systems category, three DEGs associated with the circadian rhythm-plant pathway were consistently downregulated in both the AR-FS vs AR-CK and FJ-FS vs FJ-CK comparisons (Figure 6a and b). On the other hand, in the AR-FS vs AR-CK comparison, 28 DEGs related to the plant–pathogen interaction pathway were upregulated, while 7 DEGs were downregulated. In the comparisons between FJ-FS vs AR-FS and FJ-CK vs AR-CK, numerous pathways exhibited differential regulation. In the metabolism category, 41 and 27 DEGs were upregulated, while 30 and 20 DEGs were downregulated in metabolic and biosynthesis of secondary metabolites pathways, respectively, in the FJ-FS vs AR-FS comparison (Figure 6c). Similarly, in the FJ-CK vs AR-CK comparison, 29 and 18 DEGs were upregulated in the metabolic and biosynthesis of secondary metabolites pathways, while 16 DEGs were downregulated in the biosynthesis of the secondary metabolites pathway. In the genetic information processing category, SNARE interactions in the vesicular transport pathway were exclusively downregulated in the FJ-FS vs AR-FS comparison (Figure 6c). In the environmental information processing category, the phosphatidylinositol signaling system pathway was upregulated, whereas the ABC transporters pathway was downregulated in the FJ-CK vs AR-CK comparison (Figure 6d). In the organismal systems category, DEGs related to the plant-pathogen interaction pathway were differentially expressed in both the FJ-FS vs AR-FS and FJ-CK vs AR-CK comparisons (Figure 6c and d).

### 3.6 qRT-PCR assessment for confirming frost resistance

In previous studies, several key signaling pathways, such as plant hormone signal transduction and the MAPK pathway, have been identified as crucial for plant responses to low-temperature stress [10,11,12]. These pathways are thought to play a significant role in how plants perceive and adapt to frost conditions. To further explore this, our study focused on assessing the expression of genes involved in these pathways.

**Figure 7:**
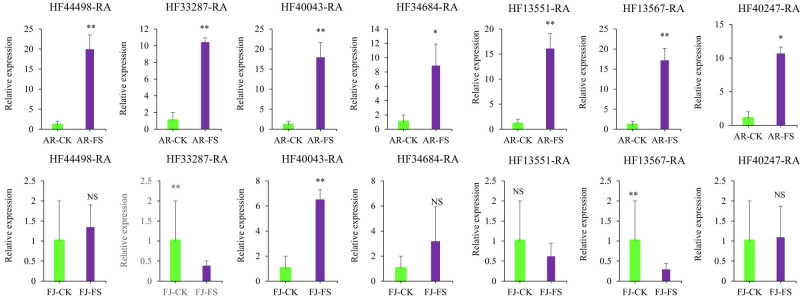
Bar graphs demonstrating the confirmation of DEGs in response to low temperature. AR-CK: “Arisoo” control, AR-FS: “Arisoo” frost stress, FJ-CK: ‘Fuji’ control, and FJ-FS: ‘Fuji’ frost stress. **p-value ≤0.01, *p-value ≤0.05, and NS signifies no significant differences. The bars depict the standard errors of the means.

We performed gene expression analysis on seven specific genes that are differentially expressed in the aforementioned signaling pathways. Our analysis, as shown in Figure 7, revealed significant differences in gene expression between frost-tolerant and frost-sensitive apple cultivars. In the frost-tolerant apple cultivar, we observed that the expression levels of all seven genes were significantly higher compared to both the control group and the frost-sensitive cultivars. This suggests that these genes may be activated as part of the plant’s adaptive response to cold stress, contributing to the enhanced frost tolerance in these cultivars. In contrast, the frost-sensitive apple cultivar displayed a different pattern. Specifically, two genes, such as HF33287-RA and HF13567-RA, showed significantly lower expression levels compared to both the control group and the frost-tolerant cultivars. Moreover, the expression of all the genes tested was generally lower in the frost-sensitive cultivar than in the frost-tolerant ones. This suggests that in frost-sensitive cultivars, the pathways associated with cold stress responses may not be as strongly activated, or they may be less efficient, leading to a reduced ability to tolerate frost stress.

## 4 Discussion

In this study, we assessed the frost tolerance of flowers from various apple cultivars. The results revealed significant differences among the evaluated cultivars, influenced by both genetic factors and environmental conditions, such as weather patterns and cultivation practices from the preceding year. This finding aligns with previous research indicating that while frost tolerance in overwintering organs is largely genetically determined, environmental factors also play a crucial role [13,14]. Over the study period, “Hongro” and “Arisoo” consistently exhibited strong flower frost tolerance, whereas ‘Fuji’ and “Honggeum” demonstrated lower tolerance. Historical records from the 2020 flowering season, when a nationwide frost spell caused widespread damage, further support these findings, as ‘Fuji’ sustained significantly greater damage than “Hongro” [15]. The results of this controlled study align with field observations, confirming the increased frost susceptibility of later-blooming cultivars like ‘Fuji.’

Considering both TFFD and KFFD rates, the cultivars showing the most pronounced genetic variation in flower frost tolerance were “Arisoo” and ‘Fuji.’ “Arisoo,” developed by the Rural Development Administration in 2013, is recognized for its excellent coloring, flavor, and resistance to anthracnose [16]. Its adoption as a viable alternative to the traditional Chuseok-season apple cultivar, “Hongro,” is increasing. In contrast, ‘Fuji,’ which dominates approximately 66% of Korea’s apple cultivation area, is valued for its superior storage capability and year-round demand. However, climate change has advanced apple blooming periods, increasing the risk of frost damage [17]. Severe flower frost damage in 2023 led to a 30% decline in domestic apple production, underscoring the vulnerability of major cultivars like ‘Fuji.’ These findings highlight the need for targeted breeding programs to develop frost-resistant apple cultivars, such as “Arisoo,” to ensure stable apple production under changing climatic conditions.

Transcriptomic analysis revealed significant differences in gene expression both within cultivars under different treatments and between cultivars under identical conditions. These findings highlight substantial variations in gene expression influenced by both treatment and cultivar-specific factors. A higher number of upregulated DEGs were observed under frost stress conditions in both cultivars, indicating the regulatory mechanisms involved in the frost stress response of apple cultivars. The GO classification and enrichment analysis illustrate the complexity of the frost stress response in “Arisoo” and ‘Fuji.’ “Arisoo” demonstrates a transcriptional response encompassing diverse biological processes, molecular functions, and cellular components, including modifications in cellular architecture and membrane trafficking. In contrast, ‘Fuji’ primarily activates regulatory pathways and catalytic activities, with enrichment in cation binding, DNA binding, and transcription regulation. A study in soybeans found that frost-tolerant genotypes exhibited upregulation of a wider range of defense mechanisms, such as osmotic adjustment and membrane stabilization [18].

KEGG pathway analysis highlights key differences in frost stress responses between the two apple cultivars while also identifying shared adaptations. Both cultivars exhibited upregulation in ascorbate and aldarate metabolism, starch and sucrose metabolism, and carotenoid biosynthesis pathways, which contribute to maintaining redox balance, energy storage, and photoprotection under frost conditions. Enhanced ascorbate metabolism plays a role in scavenging reactive oxygen species (ROS) [19], while increased activity in starch and sucrose metabolism suggests a role in osmoprotectant accumulation and energy reserves [20]. These findings align with research in maize that has identified these pathways as key components of frost stress responses [21]. In contrast, both cultivars exhibited downregulation in nitrogen and glutathione metabolism, indicating a resource shift towards pathways involved in frost tolerance.

Distinct variations in lipid metabolism were observed between the cultivars. “Arisoo” exhibited downregulation in fatty acid elongation, whereas ‘Fuji’ showed upregulation in glycerolipid and glycerophospholipid metabolism. These differences are associated with membrane lipid remodeling and fluidity maintenance, a process that has been observed in other plant species under frost stress [22]. Upregulation in plant hormone signal transduction and MAPK signaling pathways underscores their role in stress responses. Differences in these pathways indicate cultivar-specific regulatory mechanisms. Plant hormones, such as abscisic acid (ABA) and ethylene, regulate stress responses by influencing gene expression and stomatal behavior [23,24]. Downregulation of circadian rhythm-related genes suggests physiological shifts to optimize frost stress responses, a phenomenon that has been reported in previous studies [25]. Additionally, differential regulation in the plant–pathogen interaction pathway, particularly in “Arisoo,” indicates an increased activation of defense mechanisms. Previous research has demonstrated that stress conditions can prime plants for improved pathogen defense [26].

The qRT-PCR results confirm distinct differences in gene expression between the frost-tolerant and frost-sensitive apple cultivars, providing insights into the molecular mechanisms underlying frost tolerance. The elevated expression of key COR genes in the frost-tolerant cultivar aligns with findings that signaling pathways, such as plant hormone signaling and MAPK pathways, contribute to cold stress resistance. These pathways regulate protective protein activation, cell membrane stability, and stress-related gene expression [11,12]. In contrast, lower expression of these genes in the frost-sensitive cultivar suggests reduced activation of these pathways. The downregulation of HF33287-RA and HF13567-RA in ‘Fuji’ indicates differences in regulatory responses. The differential gene expression between the two cultivars highlights the role of these specific genes in cold tolerance, suggesting potential targets for improving frost resilience in sensitive cultivars.

These findings underscore the importance of specific genes and metabolic pathways in the adaptive response of apple flowers to frost stress. Identifying frost-resistant cultivars like “Arisoo” provides valuable genetic resources for breeding programs aimed at improving frost tolerance in apples. To facilitate the practical application of these findings, marker-assisted selection strategies can be developed using key frost-tolerance genes identified in this study. The genetic markers associated with frost resistance can accelerate the selection process, allowing breeders to efficiently screen for desirable traits at early developmental stages. Moreover, breeding programs can incorporate “Arisoo” as a parent in crossbreeding efforts to introduce frost-tolerance traits into commercially valuable apple cultivars without compromising fruit quality. By integrating these approaches, the development of climate-resilient apple varieties can be expedited, ensuring sustainable apple production in frost-prone regions. Future research should focus on validating these genetic markers in diverse apple populations and refining breeding strategies to maximize their effectiveness in practical applications. These efforts will strengthen the adaptability and resilience of apple orchards, ultimately supporting commercial cultivation in challenging environmental conditions.
